# Influence of gamma irradiation on rosin properties and its antimicrobial activity

**DOI:** 10.1038/s41598-023-31372-7

**Published:** 2023-03-18

**Authors:** Magd M. Badr, Ahmed Awadallah-F, Ahmed M. Azzam, A. H. Mady

**Affiliations:** 1 Polymer Laboratory, Petrochemical Division, Egyptian Petroleum Research Institute (EPRI), P.O. Box 11727, Nasr City, Cairo, Egypt; 2 Radiation Research of Polymer Department, National Centre for Radiation Research and Technology (NCRRT), Egyptian Atomic Energy Authority (EAEA), Cairo, Egypt; 3 Department of Environmental Research, Theodor Bilharz Research Institute (TBRI), P.O. Box 30-12411, Imbaba, Giza, Egypt; 4 Petrochemical Technology Laboratory, Petrochemical Department, Egyptian Petroleum Research Institute (EPRI), P.O. Box 11727, Nasr City, Cairo, Egypt

**Keywords:** Biochemistry, Chemistry, Materials science

## Abstract

The main component of rosin natural gum is abietic acid, which has an interesting chemical structure to be studied with the influence of gamma-ray and the antimicrobial activity on the properties of a cheap abundant solid state natural material of rosin. Rosin is exposed to a wide scale of gamma-ray ranges from 0 to 100 kGy. The changes in the properties are tracked by various techniques of FTIR, XRD, TGA, GPC, and SEM. The molecular weight of rosin changes from 370 g/mol to higher and the morphological properties were investigated. The irradiated rosin acid (IRA) at different irradiation doses exploited antimicrobial effect versus Gram-positive and Gram-negative as well. The inhibition zone enhanced from 15 to 33, 14 to 28, 14 to 20, and 9 to 14 mm for Gram-positive and Gram-negative, respectively. Moreover, bioactive behavior for irradiated rosin of 40 kGy recorded the highest antibacterial activity against both types of bacteria. The outcome data of antimicrobial activity are good and confirm that there is a significant effect of irradiation dose on the biocidal activity of rosin.

## Introduction

Rosin is a sustainable and natural material attained from the exudation of coniferous trees and is made up of about 90% resinous acid. Further, rosin forms different compounds such as dehydroabietic, pimaricin, labdane, and abietic acids. These compounds are strong substances versus bacteria^1^. In addition, the greatly stiff three-ring phenanthrene matrix of rosin provides the structure with outstanding mechanical features and thermos-degradation^2^. Rosin could be customized through addition and esterification processes relying on adjoined double bonds and carboxyl groups, respectively^3,4^. The attained rosin-related compounds can be inserted into the polymeric structure as a paramount matrix^5^. Lately, a huge endeavor has been made to naturally sustainable substances due to their built-in biodegradability, biocompatibility, and antibacterial activities^6^. They have two reactive sites; one being the carboxylic acid function group and the other being the hidden conjugated unsaturation center^7^. These reactive sites can be utilized for more treatment of the rosin molecule to transfer it to a monomer or different suitable intermediates to be utilized for the preparation of polymers^8^. Numerous sorts of polymers from rosin have been reported in the literature^9^. Rosin has also been polymerized or dimerized utilizing its reactive sites^10^. In addition, rosin and its related products exhibit unique properties such as biodegradability, excellent solvent dissolution, good biocompatibility, and lesser polarization in comparison with natural polymers such as chitosan, chitin, guar gum, pectin, dextrin, and others^11,12^. The utilization of mercantile rosins relies on their features, which differ related to the ratio of neutral constituents that represent 5–15%^13^. The actions of gamma-ray, X-ray, and high-energy electrons with materials initiate the ionization phenomena that conduct the generation of different ions and drive out fast-mobile electrons^14^. Moreover, the interactive reactions of gamma-irradiation from ^60^Co and X-ray photons throughout chiefly photoelectric, Compton scattering, and pair-production, the interactions of the high-energy electrons occur throughout coulombic interactions^15^. Thus, although the sorts of radiation of substances with photons or with electrons, both situations generate species of ions and electrons^16^. Thus, generated secondary, Compton, and photoelectric electrons enhance to further production of ions, the gamma-ray-produced ions suffer different chemical interactions, mostly throughout deprotonation interactions that produce the formation of free radicals^17,18^. Depending on the rate value of the dose, the existence of oxygenic gas molecules, and the existence of different antioxidants, these free radicals suffer numerous interactions^19^. In the existence of oxygenic gas molecules, whilst the irradiated with high rates of dose such as X-ray and electron beam improve the crosslinking processes of radical species, the irradiation with low-dose-rate, such as in the case of ^60^Co of gamma-ray, induces the chains degradability of throughout oxidation processes^20^. At a low dose rate, competition interactions are generated between the crosslinking chains of these free radicals and their interactions with oxygenic gas molecules^21,22^. Numerous in vitro investigations confirmed their efficiency as antimicrobials versus a wide scale of microbes^23,24^. Clinically, rosin-dependent salves demonstrated to support the recovery of infectious diseases of the skin related to injuries and ulcers^25,26^. Despite the biological activity mechanism of rosin acid not being fully clarified, the physiological results of microscopic investigations declared that exposing *Staphylococcus aureus* to rosin has significant effects on the wall thickness, cell aggregation, fatty acids constituents, and membrane potential^27^. The rising of rosin feeding is known for an augment in the microbicidal influence of rosin versus *S. aureus,* *Escherichia coli, Pseudomonas aeruginosa, Bacillus subtilis*, and *Candida albicans.* It is reported that the lowest feeding composition of rosin 10% (g/g) prohibited the development of microorganisms in the media of rosin-salve^24,28^. The antimicrobial activity of decreased gum rosin-acrylamide copolymer-based novel nano-gel elucidated 19.3–19.8 mm and 11.2–12.5 mm of inhibition zone versus S.* aureus* and *E. coli,* respectively^29^. Rosin acids-loaded polyethylene glycol-poly(lactic-*co*-glycolic acid) nanoparticles are recognized for improving the antibacterial features versus foodborne bacterial pathogens^30^. Further, rosin acid and its related nanoparticles are solidly effective versus antibiotic-resilient *S. aureus*. Formerly mode action of rosin declared that the rosin constituent damages the microbial cell wall and cell membrane as well. In electrophysiological tests, rosin exposures diminish cell membrane proton constituents in microbial cells. This behavior is related to the interruption of proton transfer in the membrane-related adenosine triphosphatase (ATPase) and leads to the uncoupling of oxidative phosphorylation. It leads to the metabolism of cells can stop and the source of energy is vanished^31^.

The key subject of this work is to investigate the influence of gamma irradiation on rosin acid properties and its antimicrobial activity. The outcome irradiated rosin acid samples with a wide range of irradiation doses are characterized by different tools such as Fourier transform infra-red, thermal gravimetric analysis, gel permeation chromatography, X-ray diffraction scanning electron microscope, transmission electron microscope, and viscometer. Further, the microbial activity of irradiated rosin was determined versus various microorganisms of Gram-positive and Gram-negative as well.

## Materials and methods

### Materials

Rosin acid was supplied from the local market (Egypt). The commercial grade rosin is provided in a solid grains form of resin obtained from conifers as reported elsewhere^32^. Tetrahydrofuran is supplied by Sigma Aldrich. Ampicillin and gentamicin were suplied from (Bio-Rad, France). All used microorganisms were obtained from the Environmental Research Department, Theodor Bilharz Research Institute (TBRI), Egypt. All reagents were analytically pure and were not further purified.

### Gamma-ray exposure

The solid samples of rosin were exposed to gamma-ray at different irradiation doses of 0–100 kGy with a dose rate of ~ 0.9 kGy/h. The source of the gamma-ray is ^60^Co as the main source using an Indian cell. The test tubes (10 ml) were filled with these powders followed by ionizing irradiation at the programmed gamma cell unit. The dose and dose rate were determined by dosimetry techniques to evaluate and estimate the accurate dose and dose rate as aforementioned. The names of coded samples are listed in Table 1.

**Table 1:** Molecular weights by GPC and weight loss (%) by TGA of R-0, R-20, R-40, R-60, R-80, and R-100 samples.

Sample	Code	Mw (g/mol)	Weight loss (%)					
			120 °C	155 °C	200 °C	240 °C	320 °C	410 °C
Rosin-0 kGy	R-0	370	1.20	4.94	13.39	39.98	72.39	89.00
Rosin-20 kGy	R-20	710	0.40	3.05	10.26	32.63	68.16	80.66
Rosin-40 kGy	R-40	719	0.06	0.70	1.63	2.60	32.47	99.77
Rosin-60 kGy	R-60	536	0.90	3.70	11.82	38.28	62.38	82.55
Rosin-80 kGy	R-80	472	1.20	3.53	9.15	27.30	60.97	79.43
Rosin-100 kGy	R-100	428	1.20	3.52	9.15	27.20	60.97	80.95

### Characterization techniques

The infrared spectra were investigated by Fourier transform infrared (FTIR) spectrophotometer, Perkin Elmer, USA, with a range of 4000–400 cm^−1^. The viscosity of a mixture of tetrahydrofuran (THF) with rosin at 25 °C is determined utilizing an Ubbelohde viscometer. The density of the mixture of THF with rosin is determined by a pycnometer with a volume of 25 ml. The pycnometer volume is calibrated as a function of temperature utilizing the Millipore Milli-Q water. Gel-permeation chromatography (GPC) (refractive index detector, empower TM2 chromatography data software, flow 1 ml/min, mobile phase THF is utilized to find out the average molecular weight. Surface morphologies of samples were carried out by scanning electron microscope (SEM), JSM-5400, JEOL Ltd., Tokyo, Japan. The thermogravimetric analysis device (TGA) is conducted utilizing a TG-50 instrument from Shimadzu (Japan) for testing the thermal decomposition of samples. The heating was carried out at a temperature range from room temperature up to 800 °C with a heating rate of 10 °C/min under a nitrogen gas atmosphere. The N_2_ gas flow was kept at a constant rate of about 20 ml/min to prevent the thermal oxidation of polymers. X-Ray Diffraction (XRD) patterns of the investigated samples were determined by an X-ray diffractometer (a Shimadzu XRD 600). XRD patterns were obtained at a scan rate of 5°/min on the diffractometer with CuK_α_ radiation source, a generator voltage of 40 kV, a generator current of 40 mA, and a wavelength of 0.1546 nm at room temperature. All the diffraction patterns were examined at room temperature and under fixed operating conditions.

### Antimicrobial activity assay

The bacterial strains Gram-positive such as *Staphylococcus aureus* and *Bacillus subtilis* and Gram-negative such as *Escherichia coli* and *Pseudomonas aeruginosa* that were previously isolated and identified based on their biochemical reactions in Environmental Research Department, Theodor Bilharz Research Institute (TBRI), from wastewater samples that collected from Qluobyia, Egypt. These bacterial strains are the most waterborne bacteria prevalent in the environment^33^. The antibacterial activity of irradiated rosin with different doses of gamma-ray (i.e., 0, 20, 40, 60, 80, 100 kGy) is estimated by modified Kirby-Bauer well diffusion technique against tested microbial species. Concisely, the pure cultures of microorganisms were sub-cultured in Müller-Hinton broth at 35 ± 2 °C on a rotary shaker at 160 rpm. For microbial growth, a lawn of culture was prepared by spreading the 100 μl fresh culture having 10^6^ colony-forming units (cfu)/ml of each test organism on nutrient agar plates with the help of a sterile glass-rod spreader. Plates were left standing for 10 min to let the culture get absorbed. Then, 6 mm wells were punched into the nutrient agar plates for testing the antibacterial activity of non-irradiated and irradiated rosin. Utilizing an automatic micro-pipette, 100 μl rosin water-suspended particles (10 mg/ml) flow onto each well on the entire plates. Then, during an all-night incubating period at 35 ± 2 °C, the various stages of the inhibition zone are determined. Antibiotic drugs of Gentamicin and Ampicillin are exploited as a positive control for Gram-negative and Gram-positive microbes, accordingly. Triplicate plates were used for each concentration and organism and the zones of inhibition were measured. Each experiment was performed in triplicate with mean values of ± SD^34^. Viable bacterial counts (VBCs) assay was evaluated for each bacterial species at different times after being treated with 0.01 mg/ml of the highest effective one of irradiated rosin, then the count of surviving each bacterial species was determined by plate count technique. The mean values ± SD of the reduction percentage of VBCs were calculated after treatment according to this formula:

$${\text{VBCs }}\left( \% \right) \, = \frac{{{\text{ time}}^{0} {-}{\text{ time}}^{{\text{x}}} }}{{{\text{ time}}^{0} }} \times 100$$

where time^0^ is the time before adding irradiated rosin and time^x^ is the contact time between bacteria and irradiated rosin^35^.

### TEM observation of treated bacterial species

Morphological changes of bacteria and mode of action of irradiated rosin were observed under TEM for determined the effects on Gram-positive *Staphylococcus aureus* and Gram-negative *Escherichia coli* cells. The bacterial cell was exposed to 0.01 g/ml irradiated rosin (40 KGy) for 24 h. After exposure, the samples were centrifuged and washed, then immersed in the solution formed from glutaraldehyde and paraformaldehyde with concentration (25%) and cacodylate level of buffer at 25 °C lasting one hour, after that passed in 1% osmium tetraoxide. Slices of 60 nm thickness were made using a diamond knife. The slices were put on copper grids and stained with uranyl acetate. In the end, the dehydrated grids were investigated under TEM (EM 208S Philips, Netherlands) at 80 kV, for studying the morphological changes^36^.

### Statistical analysis

The statistical analysis of the results was implemented by applying the ONE-WAY ANOVA and the results and data were examined and calculated by SPSS software version 20.

## Results and discussion

### Structure analysis

**Figure 1:**
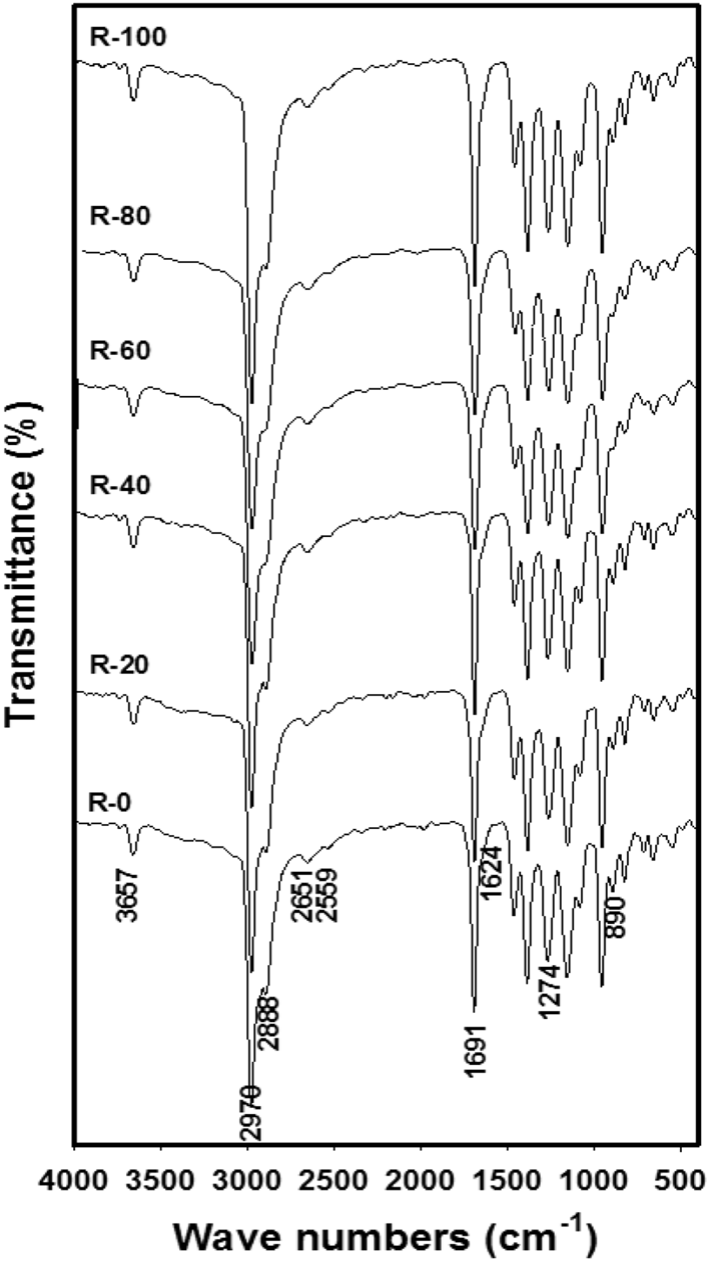
FTIR spectra of R-0, R-20, R-40, R-60, R-80, and R-100 samples at different irradiation doses and a fixed dose rate of ~ 0.9 kGy/h.

The chemical and physical rosin structure change that occurred by gamma irradiation is studied via FTIR, GPC chromatography, TGA, XRD, and SEM. Gamma-irradiated rosin forms highly viscous resin material, which is working with composites and formulations as a prepolymer for designing materials of crosslinked polymeric features and materials. FTIR spectra referring to R-0, R-20, R-40, R-60, R-80, and R-100 samples are exposed in Fig. 1. The peaks at 3657, 2651, and 2559 cm^−1^ indicate the peak of –COOH; the previous to the unbound OH (not the main band) and the final two to bonded OH, the main familiar whereas in the solid state, the carboxylic groups are likely to form dimmers^37^. These final groups come out overlapped with overtones and combination groups of lower-frequency vibrations from -COOH function groups^38^.

Stretching of -CH groups appear at 2970 and 2888 cm^−1^ also referring to a complex shape due to the existence of = CH, –CH_3_, –CH_2,_ and –CH bands, while the –CH_2_ groups display split assigned to the existence of C=C groups^39^. The narrow peak at 1691 cm^−1^ fits chiefly to C=O stretching. Further, it can be seen that a shoulder appears at 1623 cm^−1^ and overlapped a peak at 1650 cm^−1^ associated with the existence of C=C groups. The main intensity at 1274 cm^−1^ is assignable to C-O deformation from the –COOH and the band at 891 cm^−1^ is belonging to the C–H deformation out of the plane of conjugated double bonds^40,41^.

**Figure 2:**
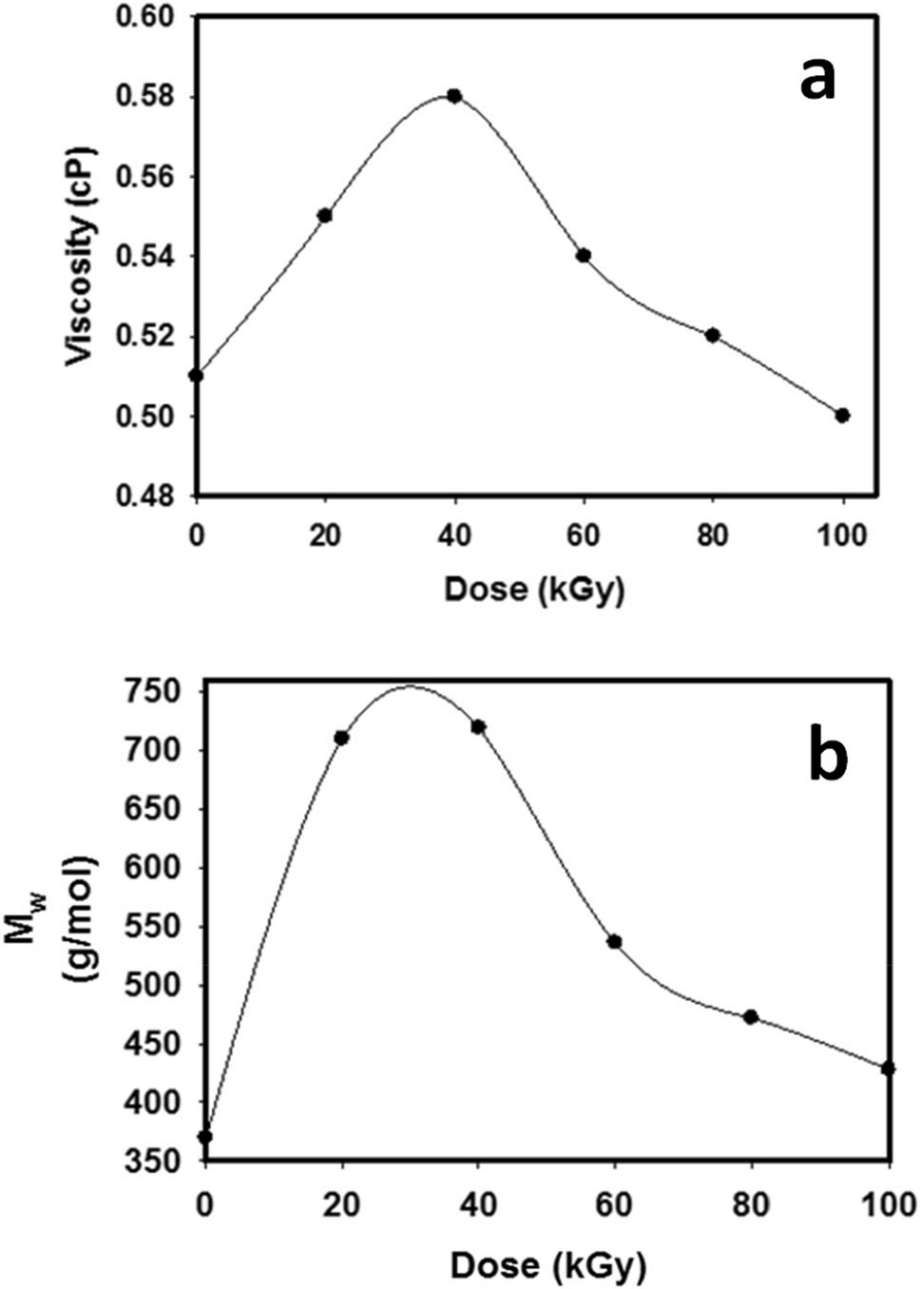
(**a**) The relationship between the viscosity of R-0, R-20, R-40, R-60, R-80, and R-100 samples and irradiation dose and (**b**) exposes the relationship between the M_w_ of R-0, R-20, R-40, R-60, R-80 and R-100 samples and irradiation dose. The fixed-dose rate of ~ 0.9 kGy/h.

**Figure 3:**
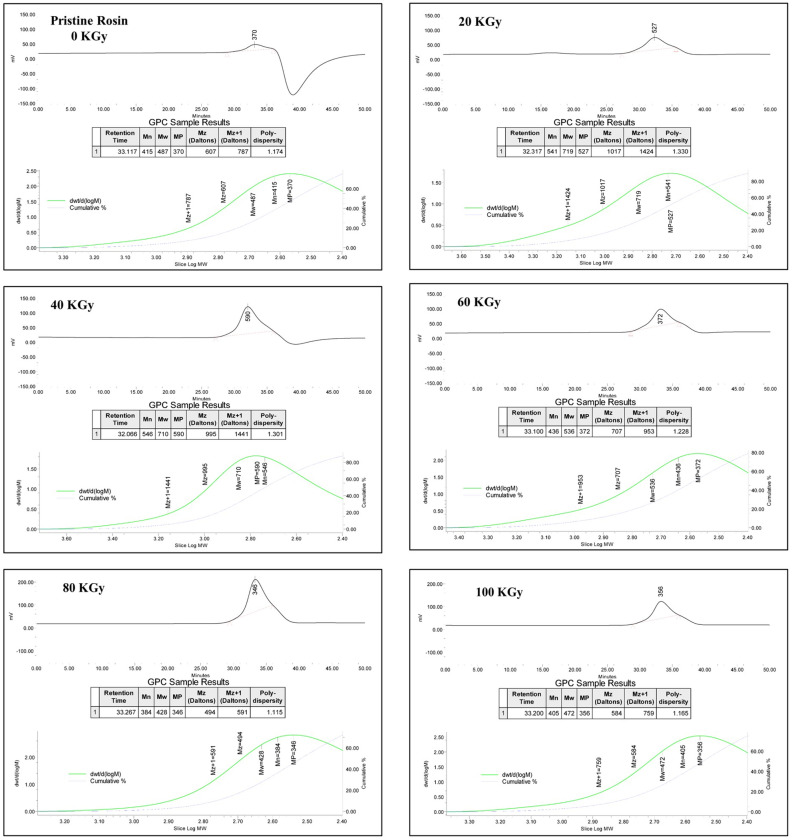
GPC charts and information of R-0, R-20, R-40, R-60, R-80, and R-100 samples.

Viscosity analysis and molecular weight measurement were expressed in Fig. 2, which shows the effect of irradiation dose on the viscosity and molecular weight of R-0, R-20, R-40, R-60, R-80, and R-100 samples, respectively. Overall, it is noticed from Fig. 2a that the viscosity augments by augmenting the dose till reaches 40 kGy and then decreases by increasing the irradiation dose. It is thought that the viscosity of rosin increases due to the cross-linking process. The decrement of viscosity of rosin after 40 kGy is due to the degradation process. The determination of molecular values of irradiated R-0, R-20, R-40, R-60, R-80, and R-100 samples at a wide range for dose is 0–100 kGy with a rate of dose ~ 0.9 kGy/h using GPC. It is seen from Fig. 2b that the M_w_ of samples augments by augmenting the dose almost near 40 kGy then augments by augmenting the dose. This may be due to partial polymerization for abietic acid up to 40 kGy then the irradiation degradation of samples after 40 kGy occurred. Further, the M_w_ values of R-0, R-20, R-40, R-60, R-80, and R-100 samples are listed in Table 1 and shown in Fig. 3.

**Figure 4:**
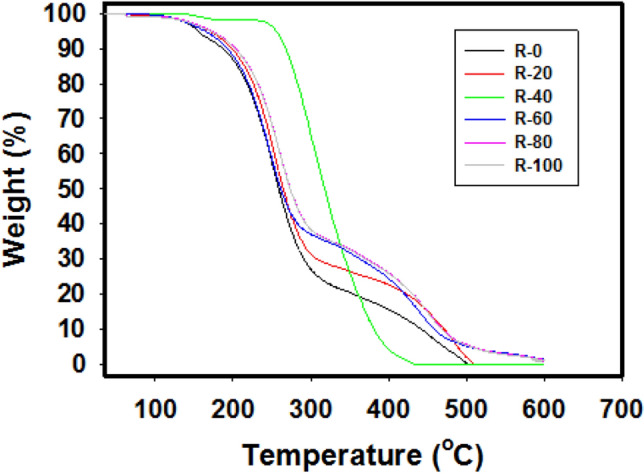
TGA thermographs of R-0, R-20, R-40, R-60, R-80 and R-100 samples with fixed rate of dose ~ 0.9 kGy/h.

Figure 4 displays the TGA thermos-curves of R-0, R-20, R-40, R-60, R-80, and R-100 samples. Overall, it is observed that by augmenting the dose, the thermal stability increases until it reaches 40 kGy for the sample of R-40 then the thermal stability decreases. The increasing thermal stability is due to the dimerization and crossing linking of abietic acid molecules, while the decrease that occurred after 40 kGy is due to the degradation of dimerized molecules of abietic acid. The dimeric constituent's thermal resistance is superior to the former situation; the dimerized constituent is perfectly formed (due to the bi-functional group existing onto abietic acid), which improved its thermal resistance^42^. Moreover, it can be noted that the R-0 has two major stages of thermal degradation, of the first stage is 320 °C and the second stage is 410 °C. The first stage is caused by the cleavage of O−O bond in 7-hydroperoxy-13-abiet-8(14)-enoic acid, and the second stage may be due to the thermal degradation of its three-ring phenanthrene skeleton^43^. From Table 1 it can be noticed that at 120–320 °C the weight loss (%) of R-40 represents the lowest value among samples, while at 410 °C represents the highest value of weight loss (%) among samples. This is due to the dimerization of the abietic aid, which increases thermal stability followed by the thermal degradation process.

**Figure 5:**
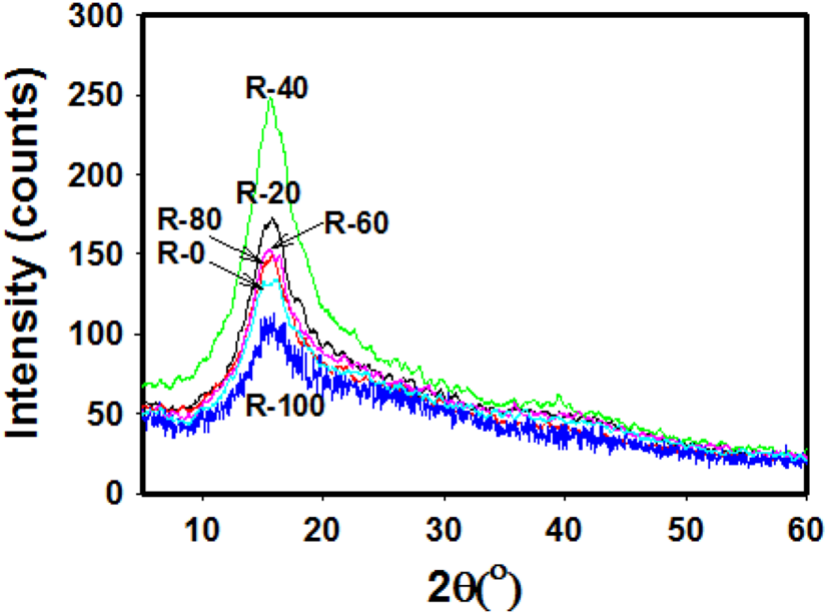
XRD patterns of R-0, R-20, R-40, R-60, R-80, and R-100 samples at different irradiation doses and a fixed dose rate of ~ 0.9 kGy/h.

The structural investigation of R-0, R-20, R-40, R-60, R-80 and R-100 samples are carried out also by XRD as depicted in Fig. 5. Through the results, the two patterns are noticed. Further, the diffractograms of specimens dehydrated at 120 °C and 150 °C are very resembling the ones mentioned in the recent study of R-0, R-20, R-40, R-60, R-80, and R-100 samples^44^. It consists of a “halo” at *2θ* = 15° that is distinctive of an amorphous structure and tends to be sharp and broad. Moreover, the small bands at *2θ* are equal to 22° and 33° proposing an extremely minute quantity of crystallinity region distributed in amorphous matrices of R-0, R-20, R-40, R-60, R-80, and R-100 samples^45,46^. Overall, it is noticed that the intensity of peak augments from 0 to 40 kGy decreases from 60 to 100 kGy. This increase in intensity is assignable to the dimerization of abietic acid, while the decrease in peak intensity may be due to some degradation structure of abietic acid according to the molecular weight of the formed structures listed in Table 1. The sequential order of peak intensity is as R-40 > R-20 R-60 > R-80 > R-0 > R-100.

**Figure 6:**
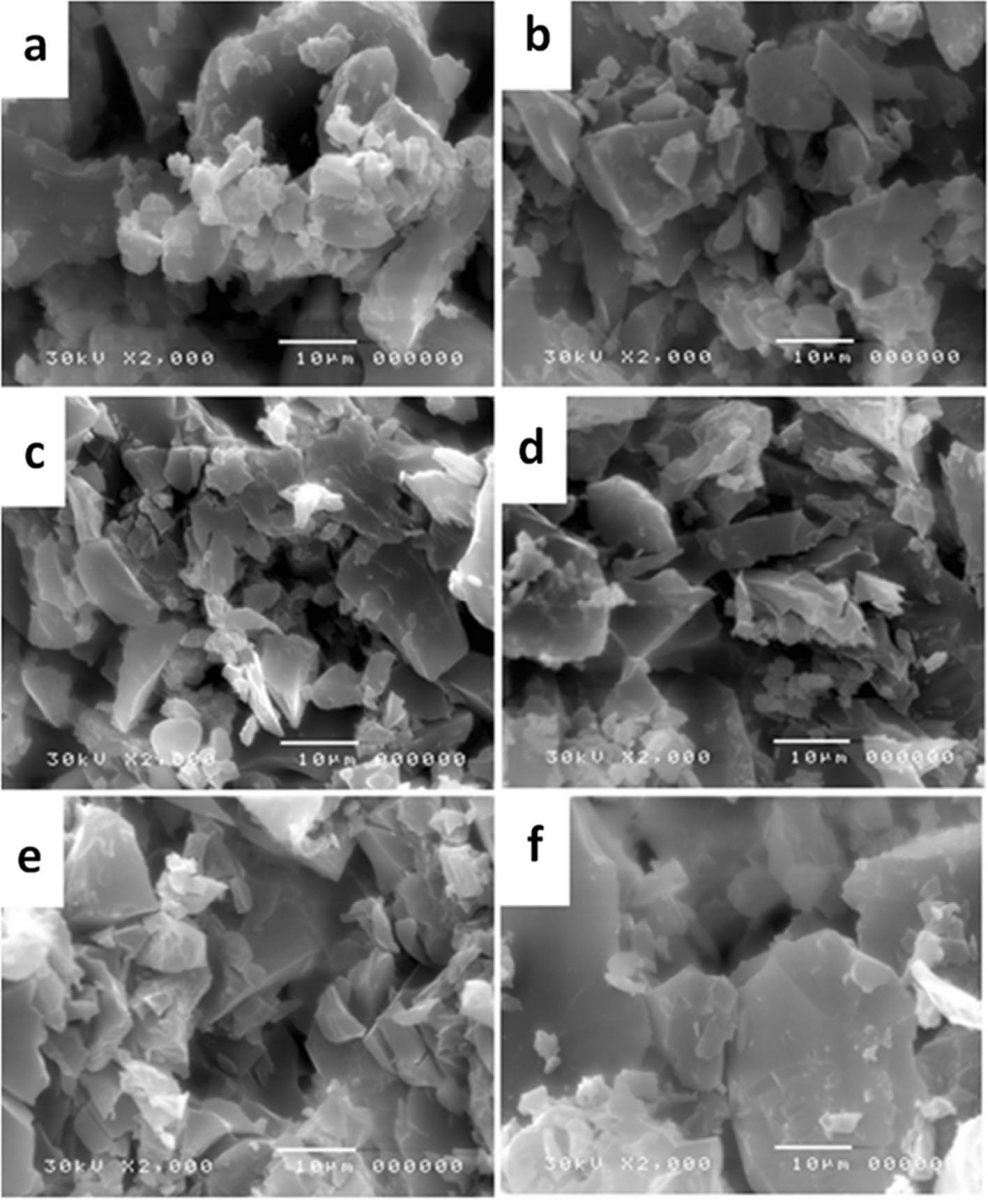
SEM photomicrographs of (**a**) R-0, (**b**) R-20, (**c**) R-40, (**d**) R-60, (**e**) R-80 and (**f**) R-100 samples with fixed rate of dose ~ 0.9 kGy/h.

To attain supplementary knowledge on surface characteristics of dehydrated R-0, R-20, R-40, R-60, R-80, and R-100 samples, their morphologies were examined by photomicrograph analysis of SEM as shown in Fig. 6. Overall, it can be observed that the photomicrographs of R-0, R-20, R-40, R-60, R-80, and R-100 samples could refer to grains and gapes structures. The homogeneity of the continuous structure was more observed with higher doses of radiation, while the gaps of inter-grains spaces of the R-0 sample are greater than the porosity between grains in R-20, R-40, R-60, R-80, and R-100. Therefore, the effect of gamma rays on R-20, R-40, R-60, R-80, and R-100 samples has a noticeable little change to some extent. It was observed that the chemical structure change that occurred by gamma radiation was combined with little physical properties change.

**Figure 7:**
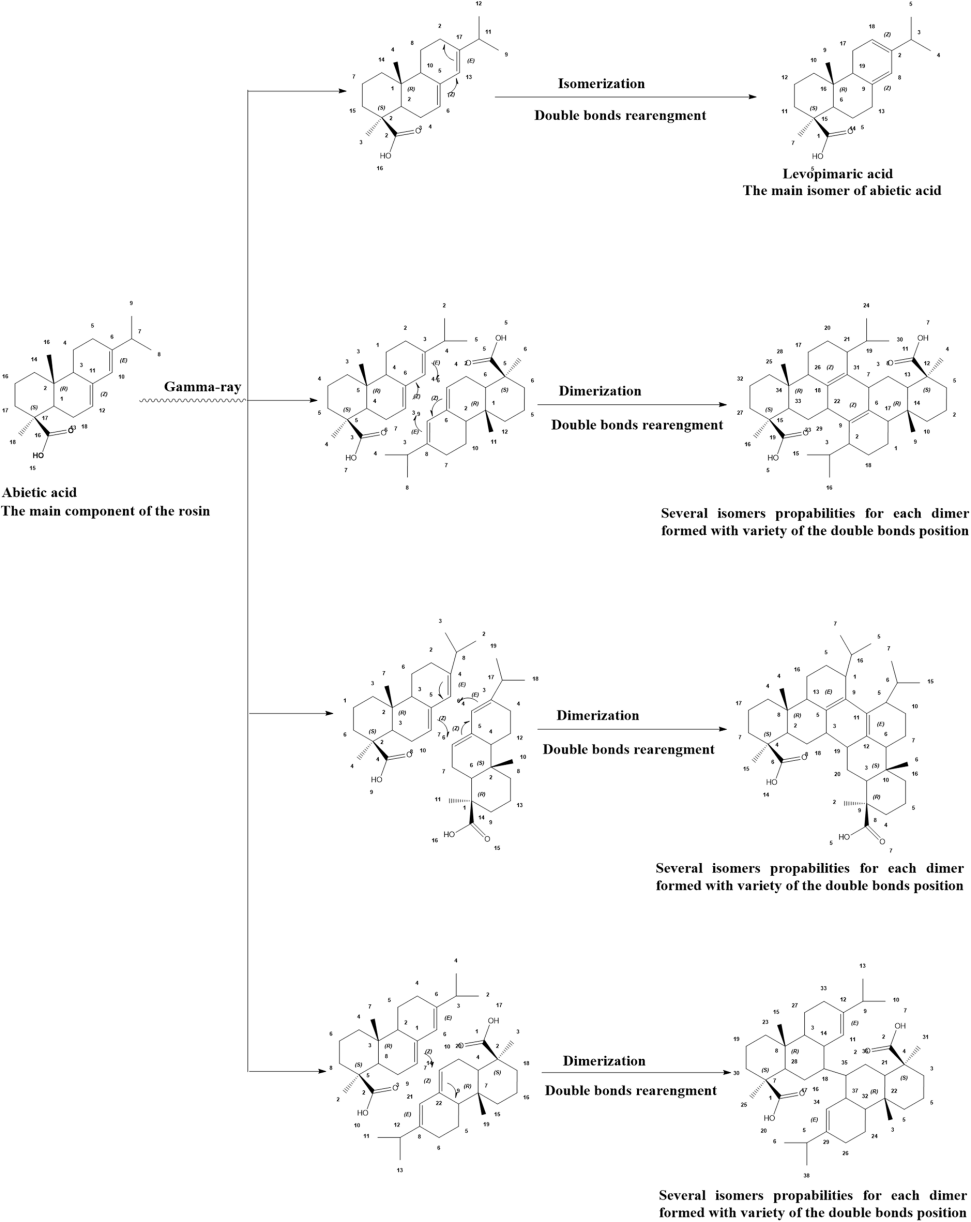
The suggested reaction mechanism of gamma-ray effect on abietic acid.

From the overall characterization, the proposed chemical reaction mechanism of the gamma-ray effect on rosin was presented in Fig. 7, while similar reactions were reported by scientists before^47,48^. The suggested reaction mechanisms were showing the double bond rearrangement of abietic acid forming the isomer levopimaric acid and several dimers.

### Antibacterial activity of irradiated rosin

#### Inhibition zone and viable bacterial counts assay

**Figure 8:**
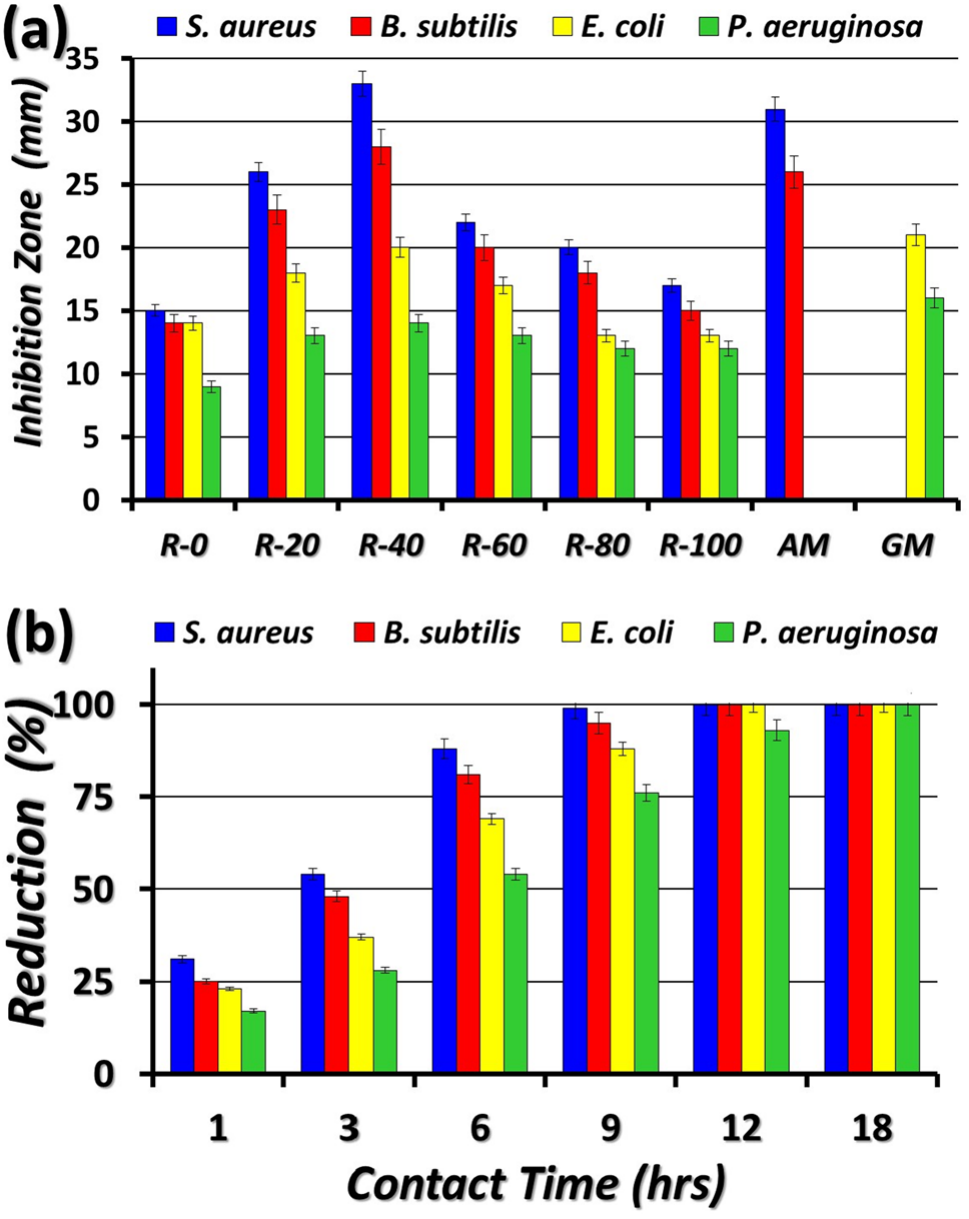
(**a**) Inhibition zones of blank and irradiated rosin and antibiotics (AM = Ampicillin and GM = Gentamicin) against four tested bacterial species, and (**b**) The reduction (%) of total viable bacterial counts (VBCs) for four tested bacterial species after exposure to irradiated rosin (R-40) at different contact times.

Figure 8a,b displays the Inhibition zone and viable bacterial counts assays, respectively. It is noteworthy to mention that Table 2 shows the inhibition zones of different types of irradiated rosin acid against the bacteria of waterborne. The inhibitions declared that irradiated rosin samples have a variable bactericidal influence on all bacteria types Gram-positive. The highest zones of inhibition were recorded with irradiated rosin (R-40), which represented 33, 28, 20, and 14 mm versus *S. aureus*,* B. subtilis*, *E. coli*, and *P. aeruginosa*, accordingly. However, by increasing or decreasing irradiation dose inhibition zones decreased as shown in Fig. 8a. The bacteria of Gram-positive are more responsive to irradiated rosin than the bacteria of Gram-negative. This result is assignable to the difference in the responsiveness of two types of microbes to irradiated rosin (40 kGy) antibacterial factor due to the dissimilarity in the contraction of the cell wall. Nevertheless, the type of Gram-positive has a simple membrane of cells that composes solely of peptidoglycan layers. On the contrary, the type of Gram-negative has a complicated wall constructed with external and internal membranes including a multi-lyres intermediate of peptidoglycan. Therefore, the wall of the cell for a positive type could be demolished more smoothly than the negative type^49^. Moreover, the assay of antibacterial killer-time for viable bacterial counts (VBCs) illustrated that the VBCs reduction percent of *S. aureus, B. subtilis, E. coli*, and *P. aeruginosa* count after being treated with irradiated rosin (R-40) at 1 h contact time were 31, 25, 23 and 17%, respectively, whereas VBCs reduction reached after 9 h to 99, 95, 88, and 76%, respectively, while complete inhibition (100%) occurred after 9 h, except for *P. aeruginosa* was at 18 h as depicted in Fig. 8b. The outcome data is in agreement with those reported in the literature^36,50^.

**Table 2:** Zone of inhibition (mm) 10 mg/ml of R-0, R-20, R-40, R-60, R-80, and R-100 samples.

Sample	Zone of inhibition (mm) 10 mg/ml			
	*S. aureus*	*B. subtilis*	*E. coli*	*P. aeruginosa*
Rosin-0 kGy (R-0)	15 ± 1.0	14 ± 1.0	14 ± 1.0	9 ± 0.5
Rosin-20 kGy (R-20)	26 ± 1.3	23 ± 1.0	18 ± 1.3	13 ± 1.3
Rosin-40 kGy (R-40)	33 ± 2.1	28 ± 1.4	20 ± 1.4	14 ± 1.3
Rosin-60 kGy (R-60)	22 ± 1.3	20 ± 1.2	17 ± 1.2	13 ± 1.0
Rosin-80 kGy (R-80)	20 ± 1.2	18 ± 1.2	13 ± 1.0	12 ± 1.0
Rosin-100 kGy (R-100)	17 ± 1.0	15 ± 1.0	13 ± 1.0	12 ± 1.0
Ampicillin^a^ (AM)	31 ± 1.5	26 ± 1.3	–	–
Gentamicin^a^ (GM)	–	–	21 ± 1.5	16 ± 1.0

^a^M_w_ of AM and GM were determined by the manufacturing company as mentioned in the experimental section. Note that each concentration of AM and GM is 10 µg and sued as standard materials.

**Figure 9:**
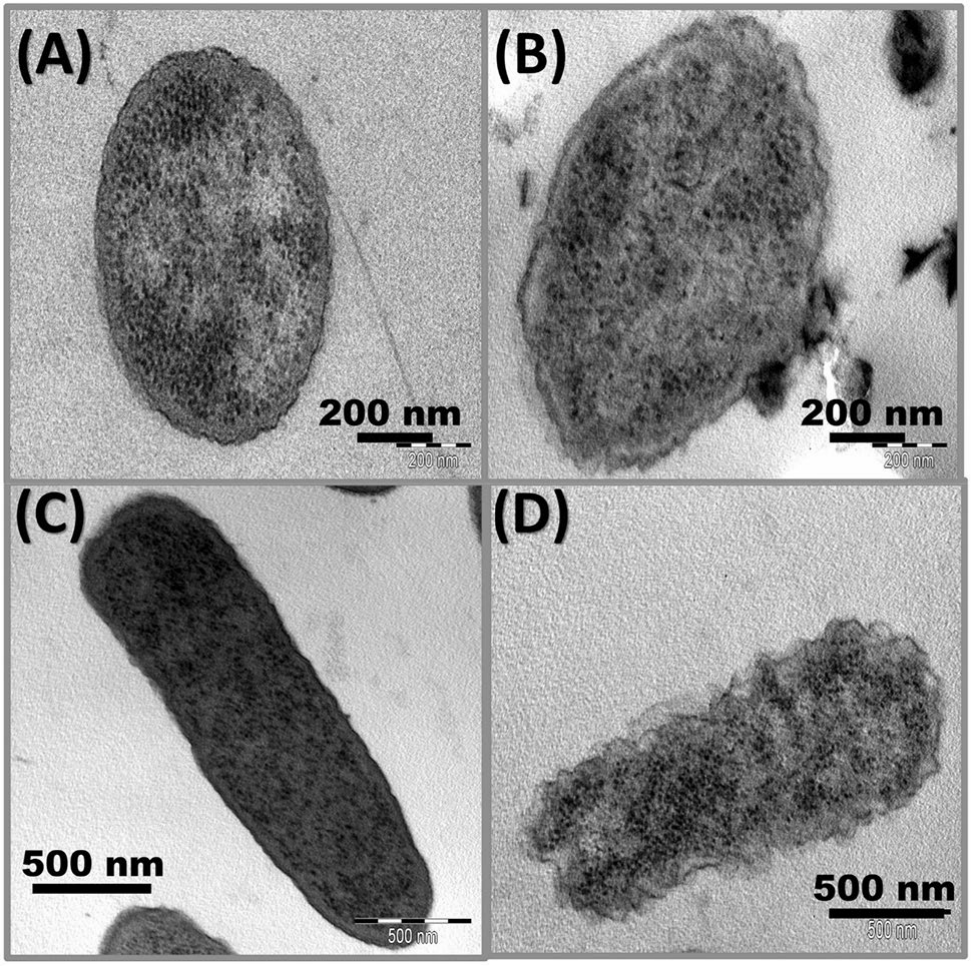
TEM images of *S. aureus* and* E. coli* in free LB medium (**A**,**C**) and after exposure to irradiated rosin of R-40 (**B**,**D**), respectively.

Figure 9 shows the TEM photomicrographs of bacteria before and after exposure to irradiated rosin (R-40) (The most effective one). The irradiated cells of bacteria exposed some changes in morphological cells and the solidity of the membrane. TEM photomicrographs confirmed that controlled bacterial cells have a solid normal cell membrane, intracellular components, and cell morphological features. Otherwise, treated bacterial cells with irradiated rosin displayed disorders in its features of morphology, which turn out to be smaller or bigger than the control. Further, the membrane of the cell was most likely and significantly demolished, and the internal cellular constituents, such as DNA, cytoplasm, and mitochondria were mostly lysis. The irradiated rosin attained in this study declared considerable antibacterial activity. The bacteria of Gram-positive are more responsive to irradiated rosin than the bacteria of Gram-negative assigned to the difference in cell wall composition. Nonetheless, the bacteria of Gram-positive have a simple cytoplasmic membrane that consists only of peptidoglycan lyres and teichoic and lipoteichoic acids. However, the cell wall of Gram-negative bacteria is more complex and has a supplementary layer formed from lipopolysaccharide and a fine layer of peptidoglycan. Thus, the external wall of bacteria for Gram-negative is more defiant to antibacterial factors. Thereby, the cell wall of the positive type can be destroyed more effortlessly than a negative one^50,51^. However, irradiated rosin that inflicts destruction to all types of cell walls of Gram types affects the inactivation of cells in *S. aureus* and *E. coli* (Fig. 9). The two main actions of the antibacterial influence of irradiated rosin are direct friction between rosin and the cells that lead to wall destruction and considerable discharge of ROS from active sites on the rosin surface. This incidence employs the action of bactericidal, causing cytotoxic effects against pathogenic bacteria^52^.

### Mechanistic antibacterial activity of irradiated rosin acid (IRA)

**Figure 10:**
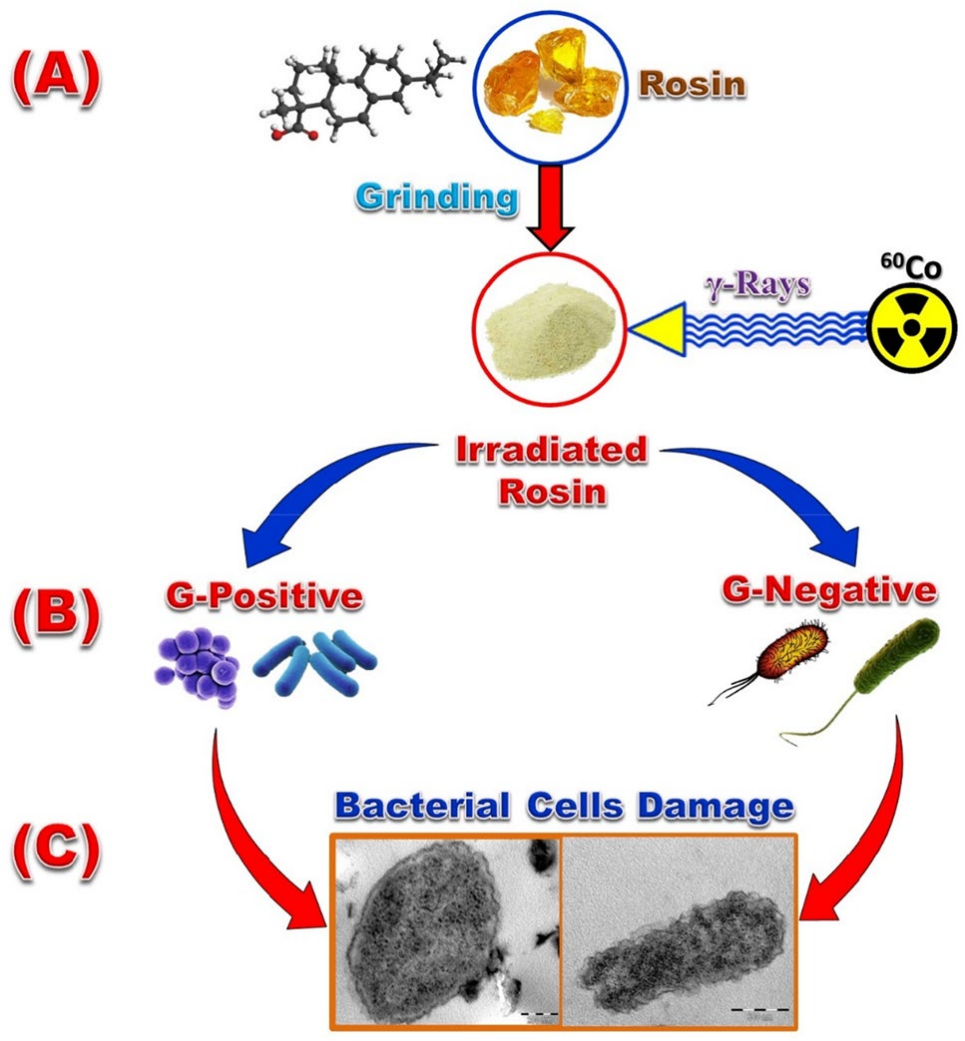
A suggested schematic diagram of (**A**) irradiation of rosin, (**B**) interaction of rosin with different kinds of bacterial and (**C**) cells of bacterial inactivated by disruption of the cell wall and lysis of intracellular constituents DNA, cytoplasm, and mitochondria.

The structural features of irradiated rosin that have reactive surface clarify the type of interaction between the irradiated rosin and bacteria that rose throughout the sequential points:

- Electrostatic interaction-induced external adsorption of irradiated rosin onto the cell membrane of bacterial^30^.
- Manufacture of ROS through irradiated rosin surface which has –COOH group.
- Extracellular and intracellular interactions between ROS produced by irradiated rosin and bacterial cell compounds.
- The cells of bacterial inactivated by the interaction between irradiated rosin and wall of cell protein employing a disturbance in portability and leakage of cellular components outside the cell and lysis of internal cellular components, such cytoplasm, DNA, and mitochondria is assignable to ROS reactions as shown in Fig. 10^53^.

## Conclusions

This study contains the influence of gamma-ray on the rosin acid properties and its antimicrobial activity. The outcome results showed that the changes in the properties of R-0, R-20, R-40, R-60, R-80, and R-100 samples occurred along the range of irradiation dose exposed. The data of FTIR, TGA, XRD, GPC, SEM, TEM, and viscosity referred to the noticeable changes in the emerged results. Moreover, the rosin acid samples at the whole range of irradiation doses showed significant antimicrobial activity versus *S. aureus*, *B. subtilis*, *E. coli*, and *P. aeruginosa*. A bioactive behavior of irradiated rosin (R-40) recorded the highest antibacterial activity against both types of bacteria, which can be attributed to ROS reactions destroying internal cellular constituents, such as DNA, cytoplasm, and ribosomes employing to the death of the bacterial cells. The irradiated rosin acid sample can provide a cheap and effective antimicrobial agent against pathogenic bacteria in the environment, which limits the problem of biological pollution and its harmful effect on human health. Moreover, it was observed that the chemical structure change that occurred by gamma radiation was combined with little physical properties change.

## Data Availability

All data generated during this study are included in this published article.

## References

[1] Söderberg TA, Gref R, Holm S, Elmros T, Hallmans G (1990). Antibacterial activity of rosin and resin acids in vitro. Scand. J. Plast. Recons..

[2] Li P, Qin L, Wang T, Dai L, Li H, Jiang J, Zhou J, Li H, Cheng X, Lei F (2020). Preparation and adsorption characteristics of rosin-based polymer microspheres for berberine hydrochloride and separation of total alkaloids from coptidis rhizome. Chem. Eng. J..

[3] Xu C-A, Qu Z, Lu M, Meng H, Zhan Y, Chen B, Wu K, Shi J (2012). Effect of rosin on the antibacterial activity against *S. aureus* and adhesion properties of UV-curable polyurethane/polysiloxane pressure-sensitive adhesive. Colloids Surf. A Asp..

[4] Feliciano AS, Gordaliza M, Salinero MA, Miguel del Corral JM (1993). Abietane acids: Sources, biological activities, and therapeutic uses. Planta Med..

[5] Li W, Cheng L, Ding M, Li W, Diao K, Liu S, Li K, Lu H, Lei F, Jiang J (2020). Rosin-based polymer@silica core–shell adsorbent: Preparation, characterization, and application to melanoidin adsorption. LWT-Food Sci. Technol..

[6] Gatenholm P, Klemm D (2010). Bacterial nanocellulose as a renewable material for biomedical applications. MRS Bull..

[7] Baldwin D, Loeblich V, Lawrence R (1958). Acidic composition of oleoresins and rosins. Ind. Eng. Chem..

[8] Chen G-F (1992). Developments in the field of rosin chemistry and its implications in coatings. Prog. Org. Coat..

[9] Cabaret T, Gardere Y, Frances M, Leroyer L, Charrier B (2019). Measuring interactions between rosin and turpentine during the drying process for a better understanding of exudation in maritime pine wood used as outdoor siding. Ind. Crops Prod..

[10] Atta AM, El-Kafrawy AF, Abdel-Rauf ME, Maysour NE, Gafer A (2010). Surface and thermodynamic properties of nonionic surfactants based on rosin-maleic anhydride and acrylic acid adducts. K. J. Dispers Sci. Technol..

[11] Duan W, Chen C, Jiang L, Li GH (2008). Preparation and characterization of the graft copolymer of chitosan with poly[rosin-(2-acryloyloxy)ethyl ester]. Carbohydr. Polym..

[12] Abeer MM, Mohd MCI, Mat LA, Manisha P, Claire M (2014). Synthesis of a novel acrylated abietic acid-g-bacterial cellulose hydrogel by gamma irradiation. Carbohydr. Polym..

[13] Gaillard Y, Mija A, Burr A, Darque-Ceretti E, Felder E, Sbirrazzuoli N (2011). Green material composites from renewable resources: Polymorphic transitions and phase diagram of beeswax/rosin resin. Thermochim. Acta.

[14] Charlesby A (1960). Atomic Radiation and Polymers.

[15] Chapiro A (1962). Radiation Chemistry of Polymeric Systems high polymers.

[16] Ferry MN, Ngono-Ravache Y, Aymes-Chodur C, Clochard MC, Coqueret L, Cortella X, Pellizzi E, Rouif S, Esnouf S, Hashmi S (2016). Ionizing radiation effects in polymers. Reference Module in Materials Science and Materials Engineering.

[17] Dole M (1972). The Radiation Chemistry of Macromolecules.

[18] Schnabel W (1981). Polymer Degradation: Principles and Practical Applications.

[19] Singh, A. Radiation Processing of Polymers. J*. Silverman. (Eds.) Hanser* (1992). https://www.bookdepository.com/Radiation-Processing-Polymers-Singh/9783446157842

[20] Ivanov VS (1992). Radiation Chemistry of Polymers.

[21] Drobny JG (2013). Ionizing Radiation and Polymers: Principles, Technology, and Applications.

[22] Ashfaq A, Clochard MC, Coqueret X, Dispenza C, Driscoll MS, Ulànski P, Al-Sheikhly M (2020). Polymerization reactions and modifications of polymers by ionizing radiation. Polymers.

[23] Li Z, Wang S, Yang X, Liu H, Shan Y, Xu X, Shang S, Song Z (2020). Antimicrobial and antifouling coating constructed using rosin acid-based quaternary ammonium salt and N-vinylpyrrolidone via RAFT polymerization. Appl. Surf. Sci..

[24] Sipponen A, Laitinen K (2011). Antimicrobial properties of natural coniferous rosin in the European Pharmacopoeia challenge test. APMIS.

[25] Sipponen P, Sipponen A, Lohi J, Soini M, Tapanainen R, Jokinen JJ (2013). Natural coniferous resin lacquer in treatment of toenail onychomycosis: An observational study. Mycoses.

[26] Sipponen A, Kuokkanen O, Tiihonen R, Kauppinen H, Jokinen JJ (2012). Natural coniferous resin salve used to treat complicated surgical wounds: Pilot clinical trial on healing and costs. Int. J. Dermatol..

[27] Sipponen A, Peltola R, Jokinen JJ, Laitinen K, Lohi J, Rautio M, Mannisto M, Sipponen P, Lounatmaa K (2009). Effects of norway spruce (picea abies) resin on cell wall and cell membrane of staphylococcus aureus. Ultrastruct Pathol.

[28] Kanerva M, Puolakka A, Takala TM, Elert AM, Mylläri V, Jönkkäri I, Sarlin E, Seitsonen J, Ruokolainen J, Saris P, Vuorinen V (2009). Antibacterial polymer fibres by rosin compounding and melt-spinning. Mater. Today Commun..

[29] Jindal R, Sharma R, Maiti M, Kaur A, Sharma P, Mishra V, Jana A (2017). Synthesis and characterization of novel reduced Gum rosin-acrylamide copolymer-based nanogel and their investigation for antibacterial activity. Polym. Bull..

[30] Santovito E, Neves J, Greco D, D’Ascanio V, Sarmento B, Logrieco AF, Avantaggiato G (2018). Antimicrobial properties of rosin acids-loaded nanoparticles against antibiotic-sensitive and antibiotic-resistant foodborne pathogens. Artif. Cells Nanomed. Biotechnol..

[31] Majeed Z, Mushtaq M, Ajab Z, Guan Q, Mahnashi MH, Alqahtani YS, Ahmad B (2020). Rosin maleic anhydride adduct antibacterial activity against methicillin-resistant Staphylococcus aureus. Polímeros.

[32] Fiebach, K. & Grimm, D. Resins, Natural. *Ullmann's Encyclopedia of Industrial Chemistry* (2000). 10.1002/14356007.a23_073

[33] Gerba CP (2015). Environmentally transmitted pathogens. Environ. Microbiol..

[34] Mohamed YM, Azzam AM, Amin BH (2015). Mycosynthesis of iron nanoparticles by Alternaria alternata and its antibacterial activity. Afr. J. Biotechnol..

[35] Azzam AM, Shenashen MA, Mostafa BB, Kandeel WA, El-Safty SA (2019). Antibacterial activity of magnesium oxide nano-hexagonal sheets for wastewater remediation. Environ. Prog. Sustain. Energy..

[36] Azzam AM, Shenashena MA, Selim MM, Alamoudi AS, El-Safty SA (2017). Hexagonal Mg(OH)_2_ nanosheets as antibacterial agent for treating contaminated water sources. Chem. Select..

[37] Flett MC (1951). The characteristic infra-red frequencies of the carboxylic acid group. J. Chem. Soc..

[38] Bratoz S, Hadzi D, Sheppard N (1956). The infra-red absorption bands associated with the COOH and COOD groups in dimeric carboxylic acid—II: The region from 3700 to 1500 cm^−1^
*Spectrochim*. Acta.

[39] Bellamy LJ (1975). The Infra-Red Spectra of Complex Molecules.

[40] Blout ER, Fields M, Karplus R (1948). Diagnosis of basal cell carcinoma by infrared spectroscopy of whole blood samples applying soft independent modeling class analogy. J. Am. Chem. Soc..

[41] Lin-Vien D, Colthup NB, Fateley WG, Grasselli TG (1991). The Handbook of Infrared and Raman Characteristic Frequencies of Organic Molecules.

[42] Prashant C, Heyu C, Shrestha RG, Ning Y (2019). Improved mechanical properties of flexible bio-based polymeric materials derived from epoxy mono/di-abietic acid and soyabean oilInd. Crops Prod..

[43] Yuanlin L, Xingliang X, Mengmeng N, Jing C, Jiahua W, Hao B, Chang Y, Min L, Li M, Fang L, Xiongmin L (2019). Thermal stability of abietic acid and its oxidation products. Energy Fuels.

[44] Lee C-M, Lim S, Kim G-Y, Kim D, Kim D-W, Lee H-C, Lee K-Y (2004). Rosin microparticles as drug carriers: Influence of various solvents on the formation of particles and sustained-release of indomethacin. Biotechnol. Bioprocess Eng..

[45] Thomas C, Nesrine H, Léo L, Jean-Bernard L, Herve M, Bertrand C (2019). A study of the physico-chemical properties of dried maritime pine resin to better understand the exudation process. Holzforschung.

[46] Takeda H, Kanno H, Schuller WH, Laurence RV (1968). Effect of temperature on various rosins and pine gum. Ind. Eng. Chem. Prod. Res. Dev..

[47] Awadallah-F A, Azzam AM, Mostafa BB, Kodous AS, Badr MM (2023). The Activity of gamma irradiated poly(Thiourea–formaldehyde) resin against aquatic microbes and cytotoxic activity. Egypt. J. Aquat. Biol. Fish..

[48] Bardyshev II, Strizhakov OD (1969). The presence of anhydrides of resin acids in rosin. Chem. Nat. Comp..

[49] Slavin YN, Asnis J, Bach H (2017). Metal nanoparticles: Understanding the mechanisms behind antibacterial activity. J. Nanobiotechnol..

[50] Doi Y, Bonomo RA, Hooper DC, Kaye KS, Johnson JR, Clancy CJ, Thaden JT, Stryjewski ME, Van-Duin D (2017). Gram-negative bacterial infections: Research priorities, accomplishments, and future directions of the antibacterial resistance leadership group. Clin. Infect. Dis..

[51] Selim MS, Azzam AM, Shenashena MA, Higazy SA, Mostafa BB, El-Safty SA (2024). Comparative study between three carbonaceous nanoblades and nanodarts for antimicrobial applications. J. Environ. Sci..

[52] Cai Y, Li C, Wu D, Wang W, Tan F, Wang X, Wong PK, Qiao X (2017). Highly active MgO nanoparticles for simultaneous bacterial inactivation and heavy metal removal from aqueous solution. Chem. Eng. J..

[53] Songfa Q, Gao F, Zhijun L, Ximing Z, Li H, Huayao C, Xinhua Z, Hongjun Z (2021). Rosin modified aminated mesoporous silica adsorbed tea tree oil sustained-release system for improve synergistic antibacterial and long-term antibacterial effects. Nanotechnolog.

